# A Matrix Metalloproteinase-2-Based Nomogram to Assess the Risk of Encapsulating Peritoneal Sclerosis in Peritoneal Dialysis Patients

**DOI:** 10.1155/2021/6666441

**Published:** 2021-01-18

**Authors:** Che-Yi Chou, Chin-Chung Tseng, Jin-Bor Chen, Chin-Chuan Hung, Chiu-Ching Huang

**Affiliations:** ^1^College of Medicine, China Medical University, Taichung, Taiwan; ^2^Division of Nephrology, Asia University Hospital, Taichung, Taiwan; ^3^Division of Nephrology and Kidney Institute, China Medical University Hospital, Taichung, Taiwan; ^4^Department of Post-Baccalaureate Veterinary Medicine, Asia University, Taichung, Taiwan; ^5^Division of Nephrology, Department of Internal Medicine, National Cheng Kung University Hospital, College of Medicine, National Cheng Kung University, Tainan, Taiwan; ^6^Division of Nephrology, Kaohsiung Chang Gung Memorial Hospital of The C.G.M.F, Kaohsiung, Taiwan; ^7^School of Pharmacy, China Medical University, Taichung, Taiwan

## Abstract

**Background:**

Encapsulating peritoneal sclerosis (EPS) is a rare but serious complication of peritoneal dialysis (PD). So far, there is no biomarker-based prediction tool available for EPS. Matrix metalloproteinase-2 (MMP-2) is a protein involved in the breakdown of the extracellular matrix, and the effluent MMP-2 can be a potential biomarker of EPS. This study is aimed at developing a nomogram for EPS based on effluent MMP-2 levels. *Patients and Methods*. We enrolled 18 EPS patients and 90 gender-matched PD patients without EPS in this cross-sectional case-controlled study. The effluent MMP-2 levels and possible risk factors for EPS were analyzed using multivariable logistic regression, and a nomogram was developed. The nomogram was validated using 200 bootstrap resamples to reduce overfit bias.

**Results:**

The effluent MMP-2 levels in EPS patients were significantly higher than those in normal PD patients (*p* < 0.001, Manny-Whitney *U* test). Effluent MMP-2 levels and PD duration were independently associated with EPS risks (*p* < 0.001 and *p* = 0.001) in multivariate logistic regression. A nomogram based on MMP-2 levels and PD duration was proposed. The AUC of MMP-2 was 0.824, and the AUC of the nomogram was 0.907 (*p* = 0.05).

**Conclusion:**

A nomogram based on effluent MMP-2 levels and PD duration may predict EPS with high accuracy.

## 1. Introduction

Encapsulating peritoneal sclerosis (EPS) is a rare but serious complication of peritoneal dialysis (PD) [1, 2]. The major features of EPS are peritoneal membrane sclerosis and intestinal encapsulation resembling a cocoon. The pathological changes of peritoneal membrane sclerosis include mesothelial denudation [3], submesothelial interstitial fibrosis, basement membrane thickening, and vascular sclerosis [4, 5]. A peritoneal membrane biopsy is needed for the diagnosis of EPS, but it is rarely performed because of its invasiveness nature. Therefore, PD effluent biomarkers may serve as noninvasive tools for detecting EPS [6]. Proteins involved in the remodeling of the extracellular matrix (ECM) are potential biomarkers for EPS because the remodeling of ECM is critical in fibrosis. Matrix metalloproteinase-2 (MMP-2) is a protein involved in the breakdown of ECM. Both animal and human studies have shown increased MMP-2 levels in peritoneal effluents of patients with EPS. MMP-2 may be a potential biomarker for EPS [7]. So far, there is no biomarker-based prediction tool available to predict EPS. This study is aimed at developing a nomogram based on effluent MMP-2 levels and other clinical parameters to identify the onset of EPS in long-term PD patients.

## 2. Methods

### 2.1. Patient Enrollment

The recruitment and follow-up protocols complied with the Declaration of Helsinki and were approved by the institutional review board of the China Medical University Hospital (CMUH104-REC2-045), and written informed consent was obtained from each individual. We enrolled 18 EPS patients from three tertiary hospitals in Taiwan from Jan. 2012 to Dec. 2017. The diagnosis criteria of EPS included bowel obstruction, ascites, and blood-stained effluent in combination with a loss of net ultrafiltration [8]; intestinal calcification resembling a cocoon in abdominal computed tomography (CT); or gross thickening of the peritoneum enclosing some or all of the small intestine in a cocoon of opaque tissue observed via laparoscopy or laparotomy. The diagnoses of EPS were confirmed by two experienced nephrologists and a radiologist. Patients without EPS were randomly enrolled from the same population as controls with a ratio of 1 : 5 and were matched for the duration of PD. All patients were free of peritonitis for at least 1 month before the effluent collection. The duration of PD was recorded from the beginning of PD treatment to the date of enrollment or the date of EPS diagnosis. Patients' biochemical data and PD effluent were recorded at either the time of enrollment or EPS diagnosis. The biochemical data recorded included serum calcium (corrected for serum albumin), phosphorus, creatinine, and albumin. The peritoneal equilibration test (PET) results were collected at sample collection in control patients and within 3 months in EPS patients. The number of episodes of peritonitis was recorded based on the review of medical records.

### 2.2. Specimen Collection and Biochemical Assays

We collected peritoneal effluents from EPS patients at the diagnosis of EPS and at the time of the peritoneal equilibrium test (PET) in control patients. The samples were collected after a 4 h dwell time and were frozen at a temperature of −80°C within 24 h. The peritoneal effluents were sent to a central laboratory for MMP-2 measurements using the Human MMP-2 ELISA kit (Invitrogen: #KHC3081) according to the manufacturer's protocol. Effluent MMP-2 levels (*μ*g/mg) were standardized for per mg/dl of total protein in the effluent. None of the dialysate MMP-2 levels was below the detection limit. There were no missing data.

### 2.3. Statistical Analysis

Data are reported as the means (standard deviations), medians (interquartile ranges, IRQ), or frequencies (percentages) where appropriate. All continuous variables were tested for normality using the skewness and kurtosis test. Data were analyzed using the *t-*test for normally distributed variables, the Mann-Whitney *U* test for nonnormalized variables, or the chi-squared test for categorical variables. We calculated the sample size using the formula provided by Riley et al. [9]. The number of cases is 27 with a 95% confidence interval and 1.8% prevalence rate [10]. Possible factors associated with EPS were analyzed using univariable logistic regression followed by multivariable logistic regression. Factors that were significantly associated with EPS in multivariable logistic regression were selected for the constitution of the nomogram for EPS. The accuracy of the nomogram was estimated using the area under the receiver operating characteristic curve (AUC). Two hundred bootstrap resamples were used for internal validation of the accuracy estimates and to reduce overfit bias. All analyses were performed using rms and pROC packages of R Statistical Software (version 3.3.2, R Foundation for Statistical Computing, Vienna, Austria). Values with *p* < 0.05 were considered statistically significant.

## 3. Results

### 3.1. Demographic and Clinical Data

EPS patients were younger (*p* = 0.032) than the control patients (Table 1). Of the 18 EPS patients, 66.7% were female, which was similar to the proportion of female control patients (56.7%; *p* = 0.602, chi-square test). The serum levels of albumin, calcium, and phosphorus were not different between the control patients and the EPS patients. The serum creatinine levels (8.74 ± 2.61 mg/dl) of EPS patients were lower than that (11.41 ± 2.82 mg/dl, *p* = 0.017) of the control patients. The percentage of patients with high transport in PET was higher in EPS patients than in control patients.

### 3.2. Analysis of MMP-2 Levels in Peritoneal Effluents

The dialysate MMP-2 levels of EPS patients were 87.36 ng/mg (IRQ, 5.67 to 179.77 ng/mg) and were significantly higher than those (median, 16.81; IRQ, 2.32–54.01 ng/mg; *p* < 0.001; Mann-Whitney *U* test) of the control patients. The effluent MMP-2 levels significantly correlated with EPS based upon univariate and multivariate logistic regressions (*p* < 0.001 and *p* < 0.001, respectively; Table 2).

### 3.3. Nomogram for EPS

**Figure 1:**
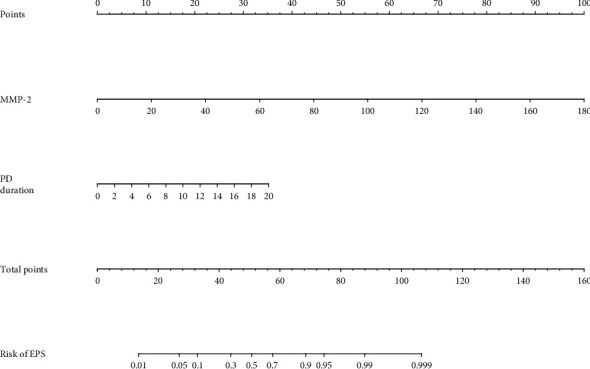
Nomogram for encapsulating peritoneal sclerosis (EPS). Points are assigned for MMP-2 levels and PD duration by drawing a line upward from the corresponding values to the “points” line. The sum of these two points, plotted on the “total points” line, corresponds to the probability of EPS by drawing a line down to the “risk of EPS” line.

**Figure 2:**
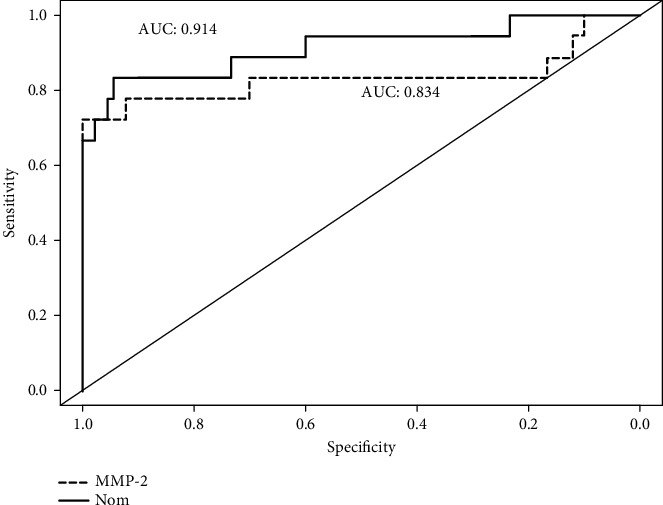
Receiver operating characteristic (ROC) curve of metalloproteinase-2 and nomogram for the diagnosis of encapsulating peritoneal sclerosis. The dotted line indicates the ROC of metalloproteinase-2, and the solid line indicates the ROC of the nomogram.

The ORs were 156 (95% CI: 18.1–1350) per log unit of MMP-2 in the univariate logistic regression and 74.7 (95% CI: 7.36–758) per log unit of MMP-2 in the multivariate logistic regression. The log-transformed MMP-2 levels were used in the logistic regressions because the distribution of MMP-2 was skewed. Longer duration of PD was associated with higher risks of developing EPS with an OR of 1.28 (95% CI: 1.03–1.60) for each additional year (*p* < 0.001) based upon the multivariable logistic regression. Patients' ages, serum phosphorus, albumin, calcium, creatinine, and PET were not associated with EPS in multivariable logistic regression and were not included in the nomogram. A nomogram including effluent MMP-2 levels and duration of PD was proposed based on the results of the multivariate logistic regression (Figure 1). The AUC of effluent MMP-2 levels was 0.824 for the diagnosis of EPS (Figure 2). The accuracy of the EPS diagnosis was further improved by adding the duration of PD (*p* = 0.05). Ultimately, the AUC of the nomogram was 0.907 for the diagnosis of EPS.

**Figure 3:**
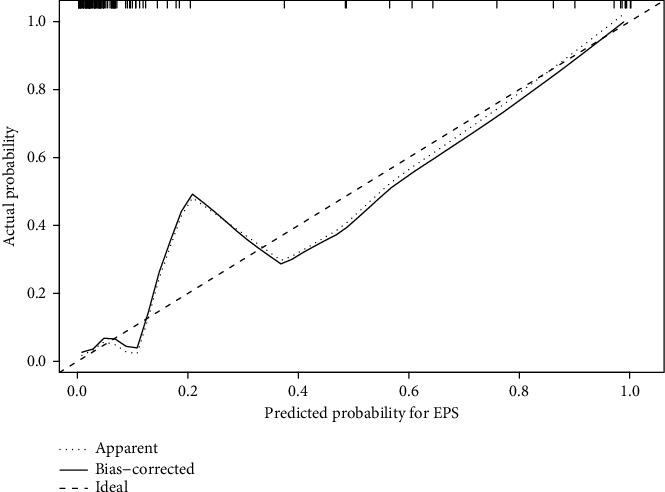
Calibration plots for the nomogram. The calibration plot shows the performance of the nomogram. Nomogram-predicted probabilities are compared to the observed rates of encapsulating peritoneal sclerosis (EPS). The *x*-axis represents nomogram-predicted probability of EPS. *y*-axis shows an observed rate of the nomogram. A perfect prediction would correspond to a slope of 1 (diagonal 45-degree broken line). The dotted line indicates the prediction of the nomogram (“apparent”), and the solid line indicates bootstrap-corrected nomogram performance.

The calibration plots are shown in Figure 3. The *x*-axis represents the nomogram predictions, and the *y*-axis represents the observed rate of EPS. The 45-degree line represents ideal predictions. The nomogram calibration plot demonstrated virtually ideal predictions. The rate of predicted nomogram closely paralleled the observed rate of EPS and nearly corresponded to the 45-degree line (Figure 3).

## 4. Discussion

This study demonstrates that an effluent MMP-2 level-based nomogram is useful for predicting the development of EPS in long-term PD patients [11, 12]. The nomogram consists of PD duration and peritoneal effluent MMP-2 levels. The use of PD duration improves the overall accuracy of EPS predictions. Increases in MMP-2 levels in the PD effluents in EPS patients may be caused by the breakdown of vascular basement membranes by MMP-2. This is because the breakdown of vascular basement membranes is the first step of neovascularization. Neovascularization is associated with increased solute transport in the peritoneal membrane [5], and MMP-2 levels are positively associated with increased solute transport in the peritoneum membrane in animal models of EPS [13]. PD duration is another component of the nomogram because longer PD duration is associated with an increase in EPS risks [1, 2, 4, 8]. It is possible that MMP-2 levels can be increased with PD duration [14, 15], but we did not find an association between MMP-2 levels and PD duration in this study.

### 4.1. Clinical Applications

This nomogram can be an important clinical tool to access the risk of EPS in chronic PD patients. If effluent MMP-2 is measured every year, a PD patient has an effluent MMP-2 level of 110 ng/mg (which corresponds to 60 points on the “points” line) and is on PD treatment for 12 years (which corresponds to 20 points on the “points” line). The total scores are 80 points that equal to 97.5% risk of EPS (0.975 on the risk of EPS axis). The physicians may decide on further investigations such as abdominal CTs, laparoscopy, holding PD treatment, and delayed removal of the PD catheter. Delayed removal of the PD catheter may facilitate the diagnosis of EPS because 45% of the patients developed EPS after stopping PD treatment [10].

The standardization of MMP-2 levels using total effluent protein levels is critical because the overall solute transport can change in EPS patients [12, 13]. MMP-2 levels can also be affected by chronic inflammation or other infections in the peritoneum, including peritonitis [8, 11, 14, 16]. Changes in solute transport may increase the total protein levels in the effluent, and some of the proteins measured in the effluent may not be specific for EPS [15, 17, 18].

The peritoneal function such as water transport can be restricted in EPS [19, 20], but we did not find an association between the results of PET and EPS in this study. Vascular calcifications are common in dialysis patients, and they are associated with all-cause mortality. The development of vascular calcifications is linked to inflammation and malnutrition in long-term PD patients [21]. However, we did not find a significant association of serum albumin, calcium, and phosphorus levels with EPS in this study. Although serum creatinine levels were significantly lower in EPS patients, creatinine levels were not associated with EPS in univariate logistic regression and were, therefore, not included in the nomogram.

There are some limitations to this study. First, some factors that may be associated with the development of EPS were not examined in this study, such as the use of PD solutions containing high concentrations of glucose. Second, markers of chronic inflammation, such as C-reactive protein or IL-6 [22], were not examined in this study. We did not analyze the association between chronic inflammation and EPS in our study. Third, the number of EPS patients was limited in this study. Thus, a larger cohort of PD patients will be needed to validate the findings of this study.

## 5. Conclusions

Higher effluent MMP-2 levels are associated with an increased risk of EPS in long-term PD patients. A simple nomogram that consists of effluent MMP-2 levels and PD duration may be associated with EPS with high accuracy.

## Figures and Tables

**Table 1:** Clinical characteristics of control and EPS patients.

Factor	Controls	EPS patients	*p*
	*N* = 90	*N* = 18	
Age (year)	59 (11)	52 (13)	0.032
Female (%)	51 (56.7)	12 (66.7)	0.602
PD duration (year)	13 [6, 20]	13 [6, 20]	—
Albumin (g/dl)	3.61 (0.36)	3.53 (0.54)	0.478
Calcium (mg/dl)	9.50 (0.81)	9.54 (0.84)	0.871
Creatinine (mg/dl)	11.41 (2.82)	8.74 (2.61)	0.017
Phosphorus (mg/dl)	4.99 (1.27)	4.34 (1.32)	0.052
MMP-2 (ng/mg)	16.81 [2.32, 54.01]	87.36 [5.67, 179.77]	<0.001

PET			
H (%)	17 (18.9)	12 (66.7)	0.001
HA (%)	37 (41.1%)	3 (16.7)	
LA (%)	7 (7.8%)	1 (5.6)	
L (%)	29 (32.2%)	2 (11.1)	
Peritonitis	1 [0, 6]	1 [0, 7]	0.12

EPS: encapsulating peritoneal sclerosis; MMP-2: matrix metalloproteinase-2; PET: peritoneal equilibration test; Peritonitis: episodes of peritonitis.

**Table 2:** Odds ratios (ORs) of possible risk factors for EPS.

Factor	Univariable	Multivariable
	OR (95% CI)	OR (95% CI)
MMP-2^∗^	156 (18.10-1350)	74.70 (7.36-758.00)
Age	0.95 (0.91-1.00)	0.96 (0.89-1.03)
Duration	1.38 (1.18-1.61)	1.28 (1.03-1.60)
Phosphorus	0.64 (0.41-1.02)	—
Albumin	0.63 (0.18-2.24)	—
Calcium	1.05 (0.56-1.98)	—
Creatinine	0.62 (0.41-1.03)	—
Peritonitis	1.32 (0.97-1.80)	—

^∗^Log transform. MMP-2: matrix metalloproteinase-2.

## Data Availability

The data are available on request.
